# NLRP3 regulates epithelial barrier integrity and protects from airway hyperresponsiveness in experimental allergic asthma

**DOI:** 10.3389/fimmu.2025.1655205

**Published:** 2025-11-27

**Authors:** Stephanie DeStefano, Ruth Grychtol, Dominik Funken, Anika Habener, Stephanie Tamm, Frauke Stanke, Wolfgang Greiner, Kirsten Beyer, Eckard Hamelmann, Michael Kabesch, Erika von Mutius, Bianca Schaub, Adan Chari Jirmo, Gesine Hansen, CHAMP study group

**Affiliations:** 1 Department of Pediatric Pneumology, Allergology and Neonatology, Hannover Medical School, Hannover, Germany; 2 Biomedical Research in Endstage and Obstructive Lung Disease (BREATH), Member of the German Center for Lung Research (DZL), Hannover, Germany; 3 Department for Health Economics and Health Care Management, School of Public Health, Bielefeld University, Bielefeld, Germany; 4 Department of Pediatric Respiratory Medicine, Immunology and Critical Care Medicine, Charité - Universitätsmedizin Berlin, Corporate Member of Freie Universität Berlin and Humboldt-Universität zu Berlin, Berlin, Germany; 5 Children’s Center Bethel, Evangelisches Klinikum Bethel, University Bielefeld, Bielefeld, Germany; 6 University Children’s Hospital Regensburg (KUNO), St. Hedwig’s Hospital of the Order of St. John and the University of Regensburg, Regensburg, Germany; 7 Institute for Asthma and Allergy Prevention, Helmholtz Center Munich, German Research Center for Environmental Health, Munich, Germany; 8 Department of Pulmonary and Allergy, Dr. von Hauner Children’s Hospital, University Children´s Hospital, Ludwig-Maximilians-University (LMU), Munich, Germany; 9 Member of the German Center for Lung Research (DZL), Comprehensive Pneumology Center (CPC-M), LMU, Munich, Germany; 10 German Center for Child and Adolescent Health (DZKJ), Dr. von Hauner Children’s Hospital, LMU, Munich, Germany; 11 Department of Pediatric Surgery, Hannover Medical School, Hannover, Germany; 12 Excellence Cluster Resolving Infection Susceptibility RESIST (EXC 2155), Deutsche Forschungsgemeinschaft, Hannover Medical School, Hannover, Germany

**Keywords:** NLRP3, Th2-inflammation, asthma, epithelial barrier, tight junction proteins

## Abstract

**Background:**

The inflammasome NLRP3 (NOD-like receptor family pyrin domain containing 3) is critical for epithelial barrier integrity. Allergic asthma is characterized by airway inflammation, airway hyperresponsiveness (AHR), and mucus hypersecretion. To date, the key players and underlying mechanisms in the interaction between NLRP3 and epithelial barrier integrity in type 2-mediated allergic asthma are poorly understood.

**Objective:**

Our study aims to evaluate the protective mechanisms of NLRP3 on airway epithelium and its structural and functional components during type 2-mediated allergic asthma inflammation.

**Methods:**

Using an experimental model of allergic airway disease, NLRP3-deficient (NLRP3^-/-^) and wild-type (WT) mice were analyzed for AHR, mucus hyperplasia, airway inflammation and the alterations in the airway epithelium transcriptome.

**Results:**

In comparison to WT mice, NLRP3^-/-^ mice exhibited significantly enhanced AHR and mucus production, while eosinophilic airway inflammation was comparable. Analysis of epithelial cell markers revealed decreased gene expression of the tight junction proteins *Cld-18* and *Tjp-1*, and decreased expression of the epithelial transmembrane protein E-cadherin in the lungs of naïve NLRP3^-/-^ mice compared to WT mice. Moreover, intranasal treatment with FITC-labelled OVA resulted in significantly higher allergen uptake by lung conventional dendritic cells (cDCs) in NLRP3^-/-^ compared to WT mice indicating increased epithelial leakiness. *In vitro*, inhibition of NLRP3 in the human bronchial epithelial cell line 16HBE14o- with MCC950 resulted in the downregulation of *Tjp-1* and *CDH1* (E-cadherin).

**Conclusion:**

NLRP3 is essential for epithelial barrier integrity in the lung and protects from the development of allergic asthma in a murine model.

## Introduction

Allergic asthma belongs to the most prevalent chronic respiratory diseases and is characterized by eosinophilic airway inflammation, mucus hypersecretion and airway hyperreactivity (1–5). In recent decades, advancements in understanding the diverse phenotypic and endotypic variations of this heterogeneous disease have facilitated the development of personalized therapeutic strategies, with a primary focus on targeting type 2 cytokines in severe eosinophilic asthma (6, 7). More knowledge is needed to improve individualized therapeutic approaches in this very common obstructive lung disease.

The inflammasome is a cytoplasmic multi‐protein complex that can be activated by various pathogen‐associated molecular patterns (PAMPs) such as lipopolysaccharide (LPS), bacterial and viral RNA or damage‐associated molecular patterns (DAMPs). The inflammasome NLRP3 (NOD‐like receptor pyrin domain‐related protein 3) is found in the cytosol of several cell types including monocytes, macrophages, dendritic cells, T and B cells, and epithelial cells (8–12). Upon activation, the inflammasome triggers the formation, production, and secretion of bioactive members of the IL-1 family, namely IL-1β and IL-18, which can initiate the inflammatory cell death pathway known as pyroptosis (13).

Several studies have shown that the NLRP3 inflammasome is involved in the development of chronic airway inflammation in asthma and COPD (14–16). However, its exact role in asthma and COPD remains controversial. Studies in mice have demonstrated that NLRP3 deficiency significantly reduces airway inflammation, immunoglobulin production, and cytokine release in response to various allergens (17–22). In contrast, other studies in different mouse models have suggested that NLRP3 may not play a significant role in acute or chronic asthma (23, 24). More recently, emerging evidence suggests that NLRP3 deficiency or deficiency of critical components of the NLRP3 inflammasome may promote the development of allergic asthma. These studies have shown increased airway inflammation, mucus secretion, and type 2 immune responses in the absence of NLRP3 or its key components (25–27).

Despite extensive research on NLRP3 in allergic airway disease, most studies have focused on its canonical inflammasome activity in immune cells and its role in promoting pro-inflammatory cytokines (28). In contrast, its function within the airway epithelium and its contribution to maintaining barrier integrity during allergic inflammation remain poorly understood. Addressing this gap is essential for understanding the broader regulatory roles of NLRP3 in airway homeostasis and for reconciling its seemingly dual function in inflammation and tissue protection.

In the healthy respiratory system, the airway epithelium acts as the first line of defense against viruses, microbes and particles as pollen by providing physical, functional, and innate immune defense protection (29, 30). It consists of ciliated and non-ciliated secretory epithelial cells, a dense mucus layer, and junctional complexes. It regulates mucus production and intercellular permeability, but also contributes to structural stability and plasticity (31–36). In allergic asthma, the epithelial barrier is compromised, resulting in increased epithelial permeability, impaired muco-ciliary clearance, and excessive mucus production (37–39).

Recent research has highlighted the critical regulatory role of NLRP3 in maintaining stability and permeability of the airway epithelium. In a mouse model of acute lung injury, NLRP3 increased adherence junctions in epithelial cells, a critical factor in maintaining airway epithelium integrity (40). Similar regulatory roles have been observed in the renal and intestinal epithelium, further highlighting the importance of NLRP3 in maintaining epithelial barrier function in other organs (41–43).

To investigate the role of NLRP3 regarding airway epithelial barrier integrity in the context of allergic disease, we compared C57BL/6 (WT) and NLRP3 knock-out (NLRP3^-/-^) mice in a well-established experimental model of allergic airway disease (44).

We found that loss of NLRP3 leads to significantly increased AHR, mucus production, and systemic IL-5 and IL-13 expression, while airway inflammation is not affected. Analysis of the epithelium of naïve unstimulated NLRP3^-/-^ mice revealed significantly reduced gene expression of the tight junction proteins *Cld-18* and *Tjp-1*, as well as reduced protein levels of the epithelial transmembrane protein E-cadherin in the lung. Allergen uptake by conventional dendritic cells (cDCs) was significantly higher in NLRP3^-/-^ mice compared to WT mice, indicating increased epithelial permeability. Furthermore, we showed *in vitro* that NLRP3 has a regulatory effect on the transcriptional expression of Tjp-1 and E-cadherin (*CDH1*) in the human bronchial epithelial cell line 16HBE14o- after inhibition of the inflammasome with MCC950.

In summary, our findings highlight a protective role of NLRP3 in maintaining the local airway epithelial barrier and controlling airway hyperreactivity and mucus hypersecretion in a Th2-mediated allergic asthma model.

## Materials and methods

### Mice

Wild-type (WT) C57BL/6J and B6.129S6-Nlrp3tm1Bhk/J (NLRP3^-/-^; C57BL/6 background) were initially purchased from Jackson Laboratory and bred and housed at the SPF animal facility of the Hannover Medical School. All experiments were approved by the animal welfare committee (Niedersächsisches Landesamt für Verbraucherschutz und Lebensmittelsicherheit; Lower Saxony State Office for Consumer Protection and Food Safety (LAVES) Permit: 17/2548). Female mice aged 8 to 12 weeks were used. Mice were sacrificed by isoflurane overdose and terminal bleeding.

### Induction of allergic airway inflammation

**Figure 1:**
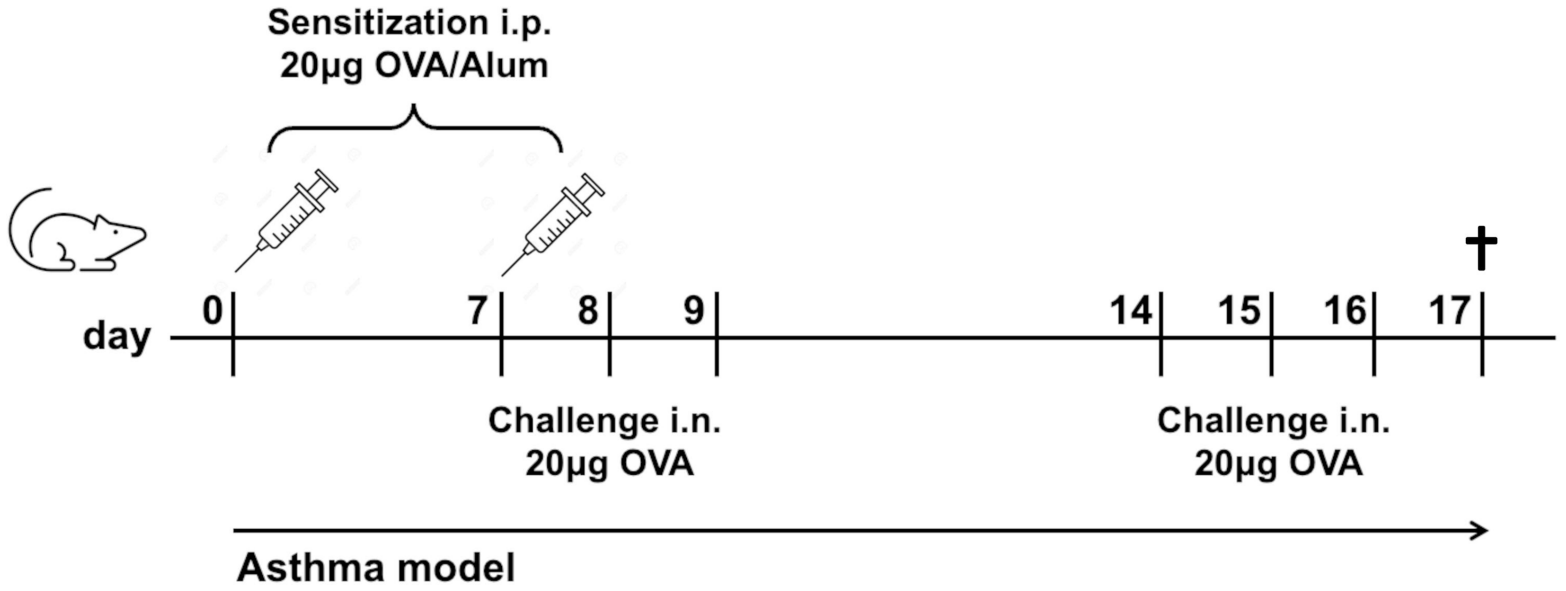
Experimental mouse model of allergic asthma. Mice were sensitized twice i.p. with OVA in Alum/NaCl 0.9% and challenged six times i.n. with OVA/NaCl 0.9%. Control mice received Alum/NaCl 0.9%, resp. NaCl 0.9% only. Mice were sacrificed one day after the last OVA challenge.

WT and NLRP3^-/-^ mice were sensitized on days 0 and 7 intraperitoneally (i.p.) with Polymyxin-treated Grade V OVA (20μg in 0.9% NaCl; Sigma‐Aldrich, Munich, Germany; LPS content reduced <20 Endotoxin Units/mg protein; LAL Test Limulus Amebocyte Lysate, Lonza, Basel, Switzerland) adsorbed to 2 mg of Alum (Inject alum adjuvant, Thermo Fisher Scientific), followed by repeated (days 7, 8, 9, and 14, 15, 16) intranasal (i.n.) challenges (20μg OVA in 40 μl 0.9% NaCl). Control mice received Alum without OVA i.p. and 0.9% NaCl i.n. instead of OVA. Lung function was measured one day after the last OVA challenge, afterwards mice were sacrificed (**Figure 1**). In some experiments, mice were sacrificed without prior lung function and/or BALF flushing.

### Measurement of lung function

Invasive lung function was assessed, as described previously (44, 45). In brief, AHR was defined as an increase in dynamic lung resistance (R) in response to increasing concentrations of MeCh (Sigma-Aldrich; 10, 20, and 30 mg/ml). One day after the last allergen challenge, mice were anesthetized with ketamine (Albrecht GmbH, Aulendorf, Germany) and xylazine (Rompun, Bayer Vital GmbH, Leverkusen, Germany), intubated, and mechanically ventilated *via* the flexiVent system (Scireq, Montreal, QC, Canada) using a tidal volume of 12 ml/kg at a frequency of 150 breaths/min. Baseline resistance was measured after exposure to 0.9% (w/v) NaCl, aerosolized in the Aeroneb nebulizer (Inspiration Medical, Bochum, Germany). Subsequently, increasing concentrations of methacholine (MeCh; 10, 20, and 30 mg/ml) were used to induce AHR. After deep inflation of the lungs, single frequency forced oscillations were performed; four peak values of resistance were analyzed for each MeCh concentration.

### Isolation of mouse primary lung cells

Lungs were digested and cells isolated as described elsewhere (46). Briefly, lungs were digested according to the respective investigation, dispase-based digestion (DispaseII; Sigma-Aldrich, Munich, Germany) for epithelial and collagenase-based digestion (Merck, Darmstadt, Germany) for all other analysis. Erythrocytes were lysed, and cells filtered through a 70 μm strainer (BD, Franklin Lakes, NJ, USA), counted, and resuspended in complete medium (RPMI1640, 10% FCS, 100 U/ml penicillin, and 100 µg/ml streptomycin (RPMI: Bio&Sell, Feucht, Germany; Pen/Strep: Sigma-Aldrich, Munich, Germany; FCS: Biochrom, Cambridge, UK).

### Analysis of bronchoalveolar lavage fluid

BALF was collected by flushing the right lung twice with 0.8 ml 2 mM EDTA in PBS. Total cells were counted using the Cedex HiRes automated analyzer (Roche, Basel, Switzerland). Differential cell counts were determined by staining cytospin preparations with May-Grünwald/Giemsa (Merck, Darmstadt, Germany) using standard microscopic criteria and counted in a blinded manner at 100× magnification (Axiovert 40 CFL, Zeiss, Oberkochen, Germany).

### Lung histopathological examination

Immediately after collection of the BALF samples, lung tissues were fixed in 4% paraformaldehyde and then embedded in paraffin before sagittal cutting into 5 μm sections. Sections were stained with HE or PAS to analyze the inflammatory cells infiltration and global cell hyperplasia, respectively. Quantification was performed as described elsewhere (47). In short, PAS-stained bronchi were scored on a scale of 0 to 4, with 0 being negative and 1–4 being positive. The quantity of mucus production in positive bronchi was scored as follows: 1, 5– 25%; 2, 25– 50%; 3, 50 – 75%; and 4, >75%. Peribronchiolar and perivascular inflammation stained with HE was scored as follows: 0, normal; 1, few cells; 2, a ring of inflammatory cells one– cell layer deep; 3, a ring of inflammatory cells two- to four-cells deep; and 4, a ring of inflammatory cells of more than four-cells deep.

### Flow cytometry analysis

Immunophenotyping of cells isolated from lung was performed using multicolor flow cytometry. Briefly, cells were blocked prior antibody staining for 15 min with a Fcγ receptor antibody (unlabeled CD16/32, clone 2.4G2; BD Biosciences, Franklin Lakes, New Jersey, USA). Exclusion of dead cells was ensured by using the LIVE/DEAD™ Fixable Aqua Dead Cell Stain Kit (ThermoFisher). Cells were stained with specific antibody panels on ice for 30 minutes (**Supplementary Table S1**) washed, resuspended in 0.1% BSA/PBS (Lonza, Basel, Switzerland), and acquired on a FACS Canto II (BD Biosciences) or CytoflexS (Beckman Coulter) flow cytometer. Data were analyzed using FlowJo software V10 (FlowJo LLC, Ashland, Oregon, USA).

### Cytokine production

Lung cells and splenocytes were re-stimulated *in vitro* (1 x 10^6^ cells/ml) with OVA (200 µg/ml) in RPMI (supplemented with 10%FCS, 100 U/ml penicillin, and 100 µg/ml streptomycin) for 72h. Cytokines were measured in supernatants using LEGENDplex™ MU Th Cytokine Panel (12-plex) (BioLegend, San Diego, USA), according to the manufacturer’s instructions.

### Measurement of OVA uptake

WT and NLRP3^-/-^ mice received a single i.n. dose of OVA-FITC (20 μg in 40 μl 0.9% NaCl; nanocs, NY, USA). Control mice received 0.9% NaCl instead of OVA. After 2h mice were sacrificed, lung cells were isolated and measured by flow cytometry. The amount of OVA internalized and processed by cDCs was quantified as FITC-positive cell frequency as well as mean fluorescence intensity (MFI).

### Gene expression analysis

Total RNA was extracted from lung cells using RNeasy Plus Mini or Micro Kit (Qiagen, Venlo, Netherlands) and reverse-transcribed with the High-Capacity cDNA Reverse Transcription Kit (ThermoFisher, Waltham, MA, USA). Quantitative PCR was performed in a 7500 Fast Real-Time PCR System (Applied Biosystems) using SYBR Green PCR Master Mix (ThermoFisher) and QuantiTect Primer Assays (Qiagen, **Supplementary Table S2**). Expression of genes was normalized to the house-keeping gene GAPDH using 2^−ΔCt^ algorithm.

### Cell culture and *in vitro* inhibition of NLRP3

Cells of the virus-immortalized human respiratory epithelial cell line 16HBE14o- (kindly provided by D. Gruenert (48)) were cultured on PureCol-coated plates (1:100; Cellsystems, Troisdorf, Germany) in MEM with 10% FCS, 1% Pen/Strep, and 1% L-Glutamine (all Thermo Fisher Scientific, Waltham, Massachusetts, USA). *In vitro* inhibition of NLRP3 was performed using the inhibitor MCC950 (Thermo Fisher, Waltham, Massachusetts, USA) at a concentration of 10µM and DMSO as mock control. In brief, cells were grown to 80% confluency, split, and seeded in petri dishes. Medium and inhibitor were exchanged every two days and finally harvested after 7 days. For RNA isolation, cells were snap-frozen in the gaseous phase of liquid N2 and stored at − 80 °C. RNA was extracted using Qiagen RNA easy Mini Kit and RNase-free DNase Set (both Qiagen, Hilden, Germany).

### Statistical analysis

Statistical significance was assessed using an unpaired two-tailed Student’s t-test, two-way ANOVA or one-way ANOVA test with Bonferroni’s multiple comparison post-test (95% confidence interval; GraphPad Prism V9.5). Results are shown as mean +/- SEM. P values less than 0.05 were considered significant [(P < 0.05 (∗), P < 0.01 (∗∗), P < 0.001 (∗∗∗), and P < 0.0001 (∗∗∗∗)].

## Results

### AHR and mucus production are significantly increased in NLRP3^-/-^ compared to WT mice in a model of allergic asthma

To analyze the impact of NLRP3 on the development of type 2-driven experimental allergic asthma, we compared NLRP3^-/-^ and WT mice in a well-established murine asthma model (**Figure 1**).

**Figure 2:**
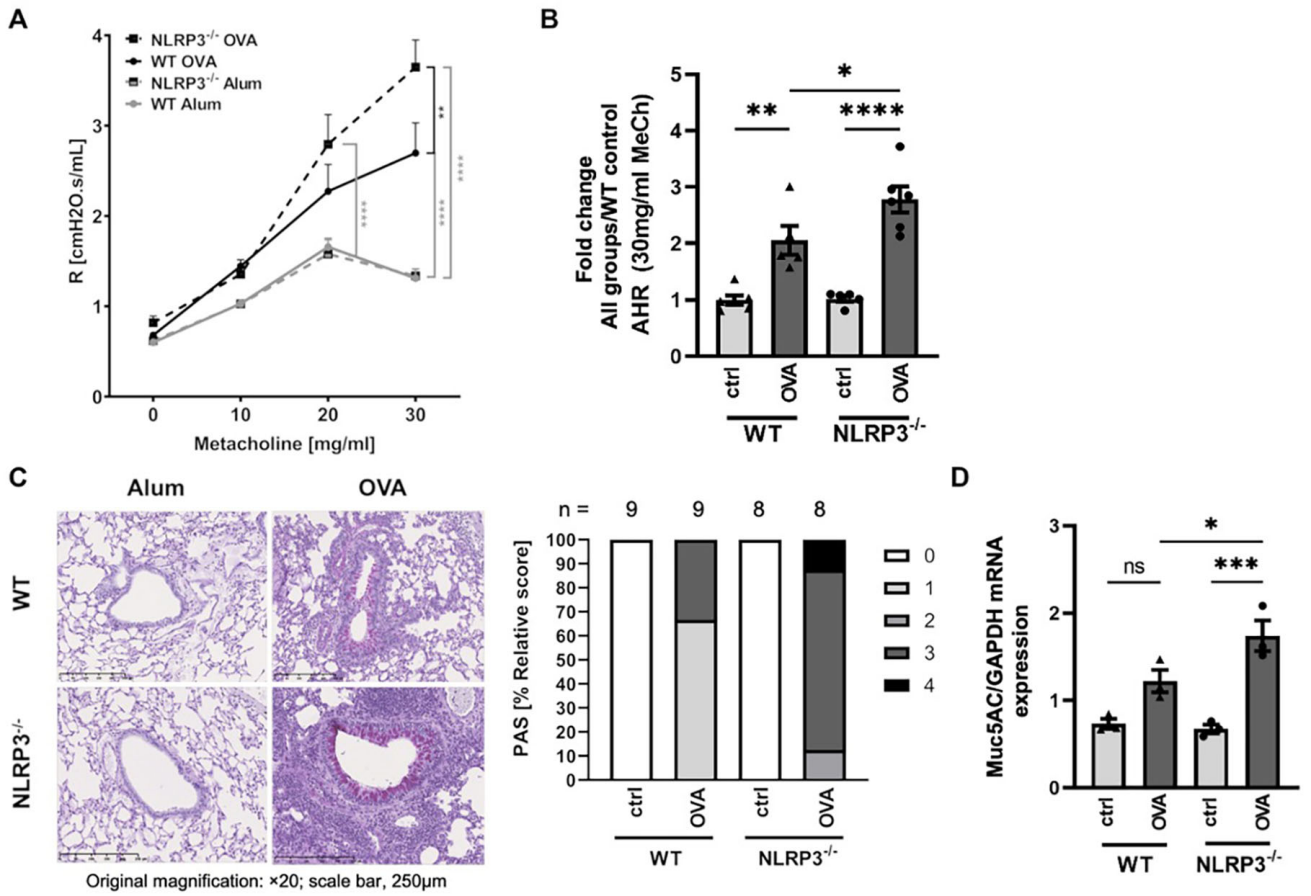
Increased AHR and mucus production in OVA-immunized NLRP3^-/-^ compared to WT mice in an experimental model of allergic asthma. **(A)** Airway resistance (R) in OVA-immunized (OVA) and control (Alum) WT and NLRP3^-/-^ mice, measured after challenge with metacholine (MeCh, n≥6/group). **(B)** Airway resistance values for 30 mg/ml MeCh as seen in A; Values are shown as fold change calculated relative to mean of WT control mice. **(C)** Levels of mucus production as determined by PAS staining of lung sections. The degree of mucus production in positive bronchi was graded as follows: 1: 5-25%; 2: 25-50%; 3: 50-75%, and 4: 75-100%. Original magnification, ×20; scale bar, 250μm; representatives of n≥8/group. **(D)** Gene expression of *MUC5AC* (n≥3/group, relative to *GAPDH*) in whole lung tissue, evaluated by RT-PCR. **(A)** Two-way ANOVA; (B&D) One-way ANOVA; mean ± SEM. ns not significant, *P < 0.05, **P < 0.01, ***P < 0.001 und ****P < 0.0001.

Following our OVA sensitization and challenge protocol (see **Figure 1**), both NLRP3^-/-^ and WT mice developed significantly increased AHR compared to their naïve control counterparts. Notably, resistance measured by invasive lung function was higher in NLRP3^-/-^ compared to WT animals (**Figures 2A, B**). Similarly, PAS staining of lung sections from immunized versus naïve NLRP3^-/-^ and WT mice revealed an increase in mucus production after OVA-immunization compared to naïve controls (**Figure 2C**). Similar to the observation of AHR, the increase in mucus production was significantly stronger in immunized NLRP3^-/-^ mice compared to WT mice. Correspondingly, measurement of gene expression of *MUC5AC*, one of the major components of airway mucus (32), which was significantly increased in both immunized WT and NLRP3^-/-^ mice, whereas it was again significantly higher in immunized NLRP3^-/-^ compared to WT animals (**Figure 2D**). Re-stimulation of immunized NLRP3^-/-^ splenocytes and lung cells with OVA revealed significantly increased systemic IL-5 and IL-13 levels in splenocyte cultures compared to immunized WT mice and a trend toward higher IL-5 and IL-13 levels in lung cell supernatants from NLRP3^-/-^ compared to WT mice (**Supplementary Figures S1D, S2**). Cytokine levels in BALF remained below the detection limit (data not shown). Our data suggest an important role for NLRP3 in lung function and mucus production regulation.

### Eosinophilic airway inflammation is comparable in immunized NLRP3^-/-^ and WT mice

In addition to increased AHR and mucus hypersecretion, allergic airway disease is characterized by eosinophilic lung inflammation. Analysis of the bronchoalveolar lavage fluid (BALF) from immunized and naïve NLRP3^-/-^ and WT mice revealed a significant influx of cells, especially eosinophils, in both immunized NLRP3^-/-^ and WT mice compared to naïve controls. In contrast to the findings for AHR and mucus hypersecretion, the eosinophilic inflammation was comparable in in NLRP3^-/-^ and WT animals after OVA immunization (**Supplementary Figure S1A**). We observed an increase in lymphocyte counts in OVA-sensitized NLRP3^-/-^ mice compared to controls. However, this difference was not statistically significant (WT OVA vs NLRP3^-/-^ OVA, p = 0.2420). The Th2 cytokines IL-4, IL-5 and IL-13 in the BALF of mice were below the detection limit. We further assessed lung inflammation in H&E-stained lung sections, which confirmed a comparable eosinophilic inflammation in OVA-immunized WT and NLRP3^-/-^ mice (**Supplementary Figures S1B, C**). IL-5 secretion, which plays an important role in eosinophil recruitment, was similar in OVA-stimulated lung cell cultures from immunized NLRP3^-/-^ and WT mice (**Supplementary Figure S1D**). Our data show that eosinophilic airway inflammation in a model of experimental allergic asthma is not mediated by NLRP3.

### NLRP3 regulates the expression of cell contact-related proteins in the airway epithelial barrier

**Figure 3:**
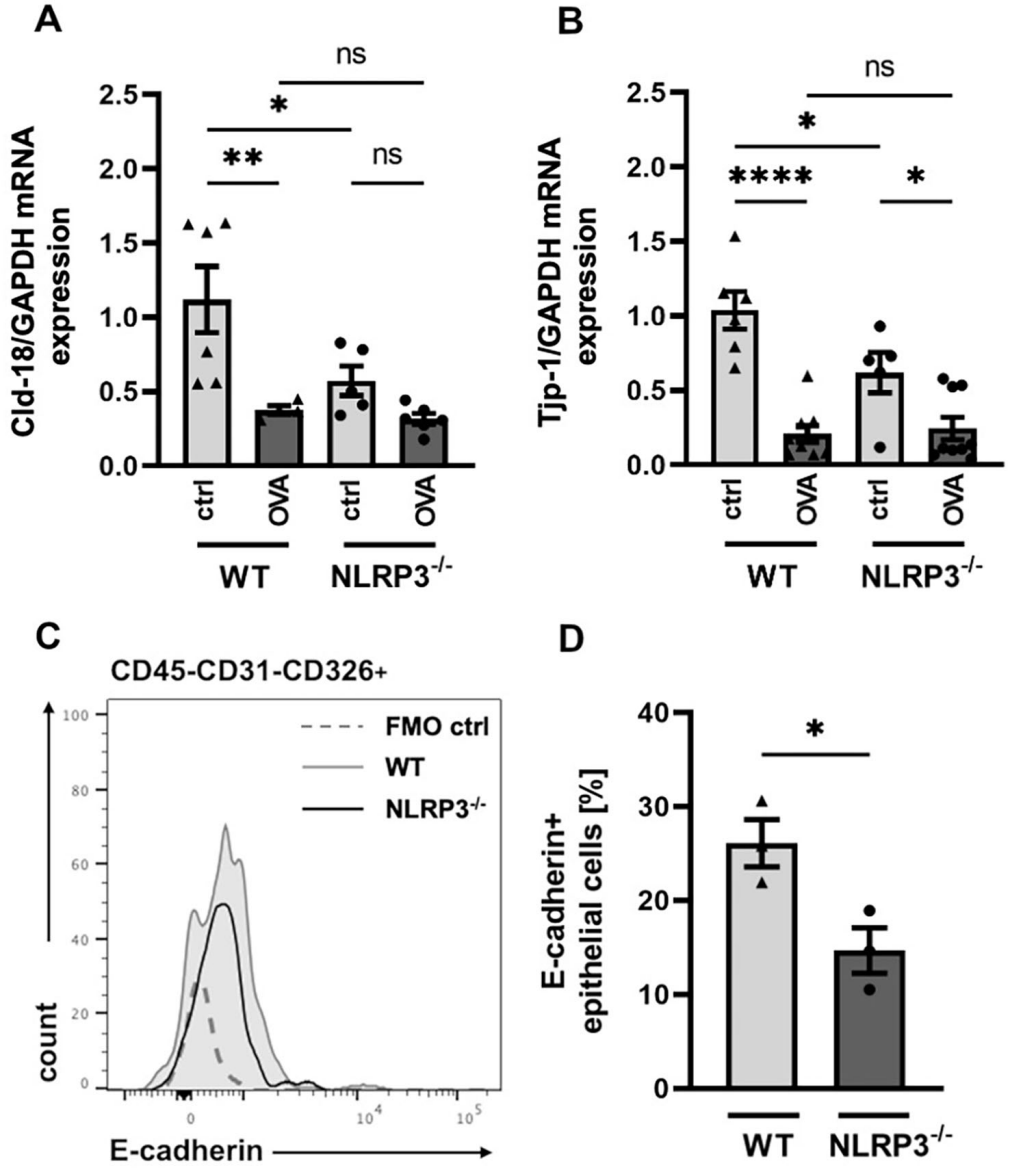
Significant differences in the expression of cell contact relevant proteins in the airway epithelial barrier of NLRP3^-/-^ and WT mice. **(A, B)** Relative gene expression of Cld-18 (Claudin-18) and the tight junction protein Tjp-1 in WT and NLRP3^-/-^ mice evaluated by RT-PCR in relation to GAPDH (n≥5/group). **(C)** Expression of E-cadherin on CD45^-^CD31^-^CD326^+^ epithelial cells isolated from lungs of WT (black line) and NLRP3^-/-^ mice (grey shaded line); (FMO: dotted line). One representative of n=3/group. **(D)** Frequency of E-cadherin+ epithelial cells in whole lung tissue of WT and NLRP3^-/-^ mice (n=3/group); unpaired two-tailed Student’s *t*-test; mean ± SEM. *P < 0.05, **P < 0.01, ****P < 0.0001.

Disruption of the epithelial layer, accompanied with enhanced epithelial permeability, is a frequently observed feature of allergic airway disease (37–39, 49). Previous studies have demonstrated the importance of intercellular cell-cell adhesion proteins for a functional epithelial barrier (50–54). Since our findings of strongly increased AHR and mucus hypersecretion in NLRP3^-/-^ compared to WT mice primarily involve changes in the epithelial barrier function, we aimed to investigate structurally relevant components, such as tight junction and transmembrane proteins, along with the efficiency of barrier integrity. Gene expression analysis of the intercellular transmembrane proteins Claudin-18 (*Cld-18)* and tight junction protein-1 (*Tjp-1*) in total lung cells showed that their expression was significantly decreased in naïve NLRP3^-/-^ mice compared to naïve WT mice (**Figures 3A, B**). Similarly, the expression of the cell-cell adhesion protein E-cadherin on CD45-CD31-CD326+ epithelial cells was significantly reduced in naïve NLRP3^-/-^ compared to naïve WT mice (**Figures 3C, D**). However, gene expression of *Cld-18* and *Tjp-1* was comparable between OVA-treated NLRP3^-/-^ and WT animals (**Figures 3A, B**).

### Conventional dendritic cells from NLRP3^-/-^ mice take up more allergen than WT mice following treatment with intra-nasal OVA

**Figure 4:**
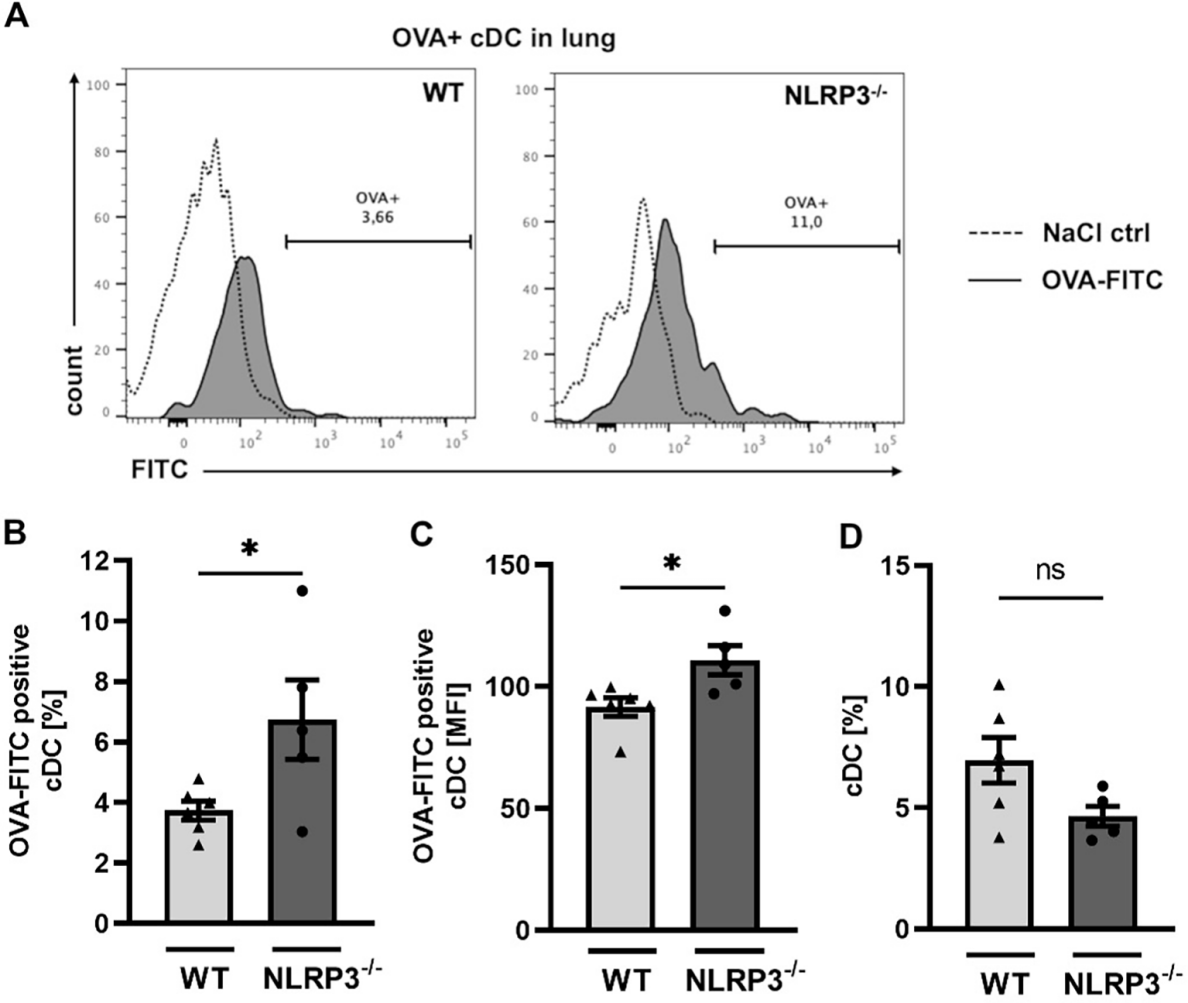
Increased OVA-uptake by conventional dendritic cells in NLRP3^-/-^ compared to WT mice. **(A-C)** OVA uptake by WT and NLRP3^-/-^ lung cDCs two hours after i.n. challenge with FITC-labeled OVA respectively. NaCl 0.9% was used as control. **(A)** Expression of FITC-OVA+ cDCs (black tinted) or control cDCs (dashed) determined by flow cytometry. Representatives of n≥5/group; **(B)** Frequencies and C mean fluorescence intensity (MFI) of FITC-OVA+ WT and NLRP3^-/-^ cDCs (n≥5/group); **(D)** Frequencies of lung cDCs in the same mice analyzed in **(B)** and **(C)**; unpaired two-tailed Student’s *t*-test; Mean ± SEM. ns not significant, *P < 0.05.

As outlined before (see **Figure 3**), we found that the gene expression of *Cld-18*, and *Tjp-1*, as well as E-cadherin protein expression was lower in NLRP3^-/-^ compared to WT mice. These genes encode proteins that are essential for an intact cell-cell contact and a functioning epithelial barrier. To test whether this dysregulation of the epithelial barrier integrity results in increased epithelial permeability for allergens, we assessed the allergen uptake capacity of the airway epithelium in WT and NLRP3^-/-^ mice. Intranasal application of FITC-labelled OVA resulted in significantly higher allergen uptake by lung resident cDCs in NLRP3^-/-^ mice compared to WT mice (**Figures 4A–C**), while the frequency of cDCs was comparable in both mouse strains (**Figure 4D**). Our results demonstrate that NLRP3 plays a critical role for the integrity of the epithelial barrier protecting against allergen uptake and possibly allergic sensitization.

### NLRP3 regulates tight junction protein and E-cadherin gene expression in human lung epithelial cell line 16HBE14o-

**Figure 5:**
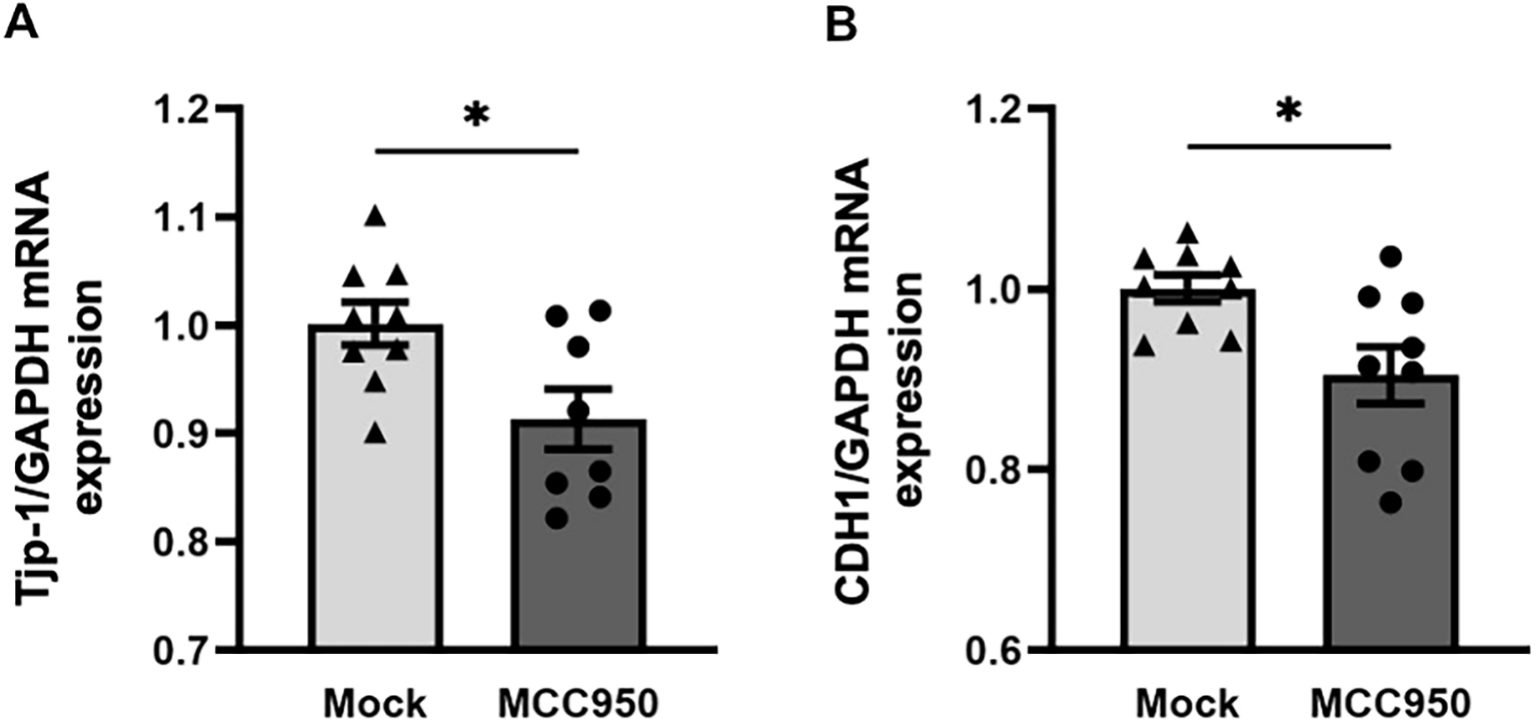
Application of the NLRP3 inhibitor MCC950 reduces the gene expression of tight junction protein 1 and E-cadherin in the human bronchial cell line 16HBE14o-. **(A)** Relative gene expression of the tight junction protein Tjp-1 and **(B)** the transmembrane protein CDH1 (E-cadherin) in cell lysates of the human bronchial cell line 16HBE14o- before and 7 days after treatment with the NLRP3 inhibitor MCC950 or DMSO. Expression was evaluated by RT-PCR in relation to GAPDH (n=9/group); Mock: DMSO, NLRP3 inhibitor: MCC950; unpaired two-tailed Student’s t-test; mean ± SEM. ns not significant, *P < 0.05.

Having observed altered gene expression of proteins important for cell-cell contact in the lungs of NLRP3^-/-^ mice, we tested our observation in a human lung epithelial cell line to see whether we could find evidence for a similar effect in humans. We added the NLRP3 inhibitor MCC950 to 16HBE14o- epithelial cells and analyzed gene expression of *Tjp-1* and E-cadherin (*CDH1*). One week after continuous application of MCC950, both genes were downregulated in MCC950-treated human epithelial cells compared to mock-treated cells (**Figures 5A, B**). This finding suggests that NLRP3 has a regulatory and stabilizing effect on the expression of cell-cell contact-related proteins in mice and humans.

## Discussion

This study examined the role of the inflammasome NLRP3 in type 2 airway inflammation using OVA-sensitized NLRP3^-/-^ and WT mice. Compared to WT mice, NLRP3^-/-^ mice displayed significantly increased airway hyperresponsiveness (AHR), mucus production, and elevated systemic levels of IL-5 and IL-13. Naïve NLRP3^-/-^ mice showed reduced expression of tight junction-associated genes and E-cadherin protein, indicating inherent epithelial barrier dysfunction. Functional assays revealed increased allergen uptake in NLRP3^-/-^ mice, linking barrier disruption to the observed phenotypic changes. However, eosinophilic airway inflammation remained similar across genotypes, suggesting that this aspect of asthma pathophysiology is independent of NLRP3 expression. These observations contrast with prior studies, underscoring the context-dependent effects of NLRP3 in type 2 airway inflammation.

Previous studies investigating the specific roles of the NLRP3 inflammasome in asthma pathogenesis have utilized various murine asthma models, yielding inconsistent results. NLRP3 has been shown to have detrimental, protective, or negligible effects on AHR, airway remodeling, and type 2 immune responses (14–25). In models using OVA, NLRP3^-/-^ mice mostly demonstrated significantly reduced inflammation and airway hyperresponsiveness compared to WT mice. Ritter et al. highlighted that NLRP3-deficient mice showed diminished levels of IL-1β, a crucial cytokine produced via the NLRP3 pathway (20). This indicates the pathway’s involvement in mediating the inflammatory response during acute allergic airway inflammation. Liu et al. also found reduced eosinophilic inflammation in NLRP3-deficient mice, primarily affecting the Th2 cytokine profile within the airways. This finding reinforces the idea that NLRP3 plays a pivotal role in promoting airway inflammation (22). However, Allen et al. showed that NLRP3 had no significant effect on airway hyperresponsiveness in allergic mice demonstrating that even in similar OVA challenge models results with NLRP3^-/-^ mice have been inconsistent (23). This variability can be attributed to differences in mouse strains, host microbiota composition, and experimental methodologies, including allergen administration protocols, preparation techniques, and timing (23, 24, 55). Contrasting findings emerged in the context of neutrophilic asthma models induced by house dust mite (HDM). Studies have shown that inflammation can still occur through different pathways even in the absence of NLRP3, and that the innate immune response remains intact. Specifically, Tan et al. reported that, although NLRP3 inflammasome inhibition effectively mitigated HDM-induced inflammation, this did not result in the complete abrogation of asthma-like symptoms. These differential responses emphasize the redundant mechanisms that can compensate for NLRP3 deficiency, particularly in models that do not produce a classic Th2 response but instead exhibit a neutrophilic phenotype (56). The results obtained from different experimental setups partly illustrate the differences in experimental design previously outlined and underscore the need for standardized approaches to clarify the role of NLRP3 in airway inflammation. Furthermore, they imply that NLRP3 plays a pivotal role in asthma development, with its effects being partially reflective of the various underlying immune mechanisms of this heterogeneous disease, and that targeting NLRP3 could lead to different therapeutic outcomes depending on the asthma subtype. Further research is needed to decipher the multiple NLRP3-related pathways with the aim of tailoring inflammasome-targeting therapeutic strategies to individual asthma phenotypes.

Our data reveal an unexpected phenotype in naïve NLRP3^-/-^ mice, suggesting a baseline defect in epithelial barrier integrity independent of inflammatory stimuli. This was not the primary focus of our study but emerged as a robust and reproducible observation. In the context of findings from other models and tissues, our data underscore a broader, homeostatic role for NLRP3 in maintaining epithelial stability. Notably, the epithelial barrier defect observed in NLRP3^-/-^ mice may reflect a combination of canonical inflammasome-dependent and inflammasome-independent functions of NLRP3. While the activation of the NLRP3 inflammasome and subsequent release of IL-1β and IL-18 release have been linked to epithelial repair and barrier maintenance, accumulating evidence suggests that NLRP3 also operates beyond its classical inflammasome role. For instance, it can directly modulate TGF-β/Smad signaling or act as a nuclear regulator of IL-33 expression in epithelial cells, thereby affecting cell adhesion, tight-junction organization, and overall epithelial stability independently of caspase-1 or IL-1β/IL-18 (57–59). Distinguishing these pathways will be crucial when considering NLRP3-targeted strategies in asthma, since barrier-stabilizing effects may not solely reflect inflammasome inhibition but also loss of homeostatic epithelial functions of NLRP3. To further support this concept, evidence from other organ systems underscores the importance of NLRP3 in maintaining epithelial barrier integrity beyond the lung.

Beyond the variability in experimental factors, studies on gastrointestinal diseases highlight a role for NLRP3 in maintaining epithelial barrier integrity, which may also be relevant to the airways. In conditions such as Crohn’s disease, ulcerative colitis, renal disease, as well as infections with pathogens NLRP3 and its downstream cytokine IL-1β are essential for preserving intestinal epithelial structure, promoting barrier repair, and ensuring tissue homeostasis (41–43, 60, 61). Similarly, research by Kostadinova et al. demonstrated that NLRP3 expression in myeloid cells enhances alveolar epithelial integrity by promoting cellular adhesion in a *Streptococcus pneumoniae*-induced pneumonia model (40). These findings suggest that NLRP3 may play a broader role in epithelial barrier maintenance across organ systems, including the respiratory tract.

Epithelial barrier dysfunction is closely associated with dysregulated airway resistance and excessive mucus production, with epithelial damage correlating with AHR severity (37–39). The airway epithelium, composed of tightly interconnected monolayers, acts as a first-line defense against pathogens while maintaining innate immunity and tissue homeostasis in the lung and intestinal tract (62–67). Increased epithelial permeability has been linked to the downregulation of E-cadherin, a key cell-cell adhesion molecule, and the disruption of tight junction complexes, which are anchored to the actin cytoskeleton, such as Tjp-1 and claudin-18 (49, 54, 68). Their integrity is closely tied to the expression and functional activity of E-cadherin and is essential for regulating molecular diffusion across tissues and maintaining epithelial barrier function (51, 53, 68, 69). These fundamental mechanisms of epithelial barrier regulation provide the context to interpret the intrinsic defects observed in the naïve NLRP3^-/-^ mice in our study.

Our findings of significantly reduced expression of tight junction-related genes and E-cadherin protein, coupled with increased allergen uptake in naïve NLRP3^-/-^ mice, suggest an intrinsic compromise in epithelial barrier integrity in these mice. The overall low expression of tight junction (TJ) genes observed in OVA-sensitized and challenged WT and NLRP3^-/-^ mice likely reflects allergen-induced epithelial barrier disruption, exacerbated by inflammatory cytokines and cellular stress responses (56, 70, 71). While Besnard et al. reported impaired DC recruitment to the draining lymph nodes in NLRP3-deficient mice, our OVA-FITC analysis in the lung did not reveal a major reduction of cDCs, making it more likely that the enhanced antigen signal we observed reflects increased epithelial leakiness rather than differences in DC antigen uptake capacity (19). Furthermore, IL-13 has been shown to impair TJ integrity in airway epithelial cells, as demonstrated by Wise et al., supporting our finding of significantly elevated IL-13 levels in both WT and NLRP3^-/-^ mice following OVA sensitization (72).

Previous research has shown that TJs are critical for maintaining epithelial barrier integrity by regulating ion and solute flux while preventing uncontrolled mucin and protein efflux, particularly from goblet cells. In our NLRP3^-/-^ mice, TJ disruption likely increases mucosal permeability, compromising selective barrier function and leading to excessive mucus secretion (52, 73–75). Therefore, our findings align with previous studies and suggest an important role of NLRP3 in the expression of TJs and, therefore, the maintenance of epithelial barrier function and regulating mucus production in response to inflammatory stimuli.

To investigate the cellular and molecular mechanisms governing airway tight junctions, epithelial barrier properties, and mucus production, immortalized human bronchial epithelial cell lines such as 16HBE14o- have been extensively utilized (76, 77). These cells, which exhibit characteristics of superficial epithelial cells, were originally derived through viral transformation of cells from human bronchial epithelium. We observed that treatment of 16HBE14o- cell monolayers with the highly specific NLRP3 inhibitor MCC950 (78–80) led to a significant reduction in the expression of *Tjp-1* and *CHD1* genes, further emphasizing the critical role of NLRP3 in maintaining cell-cell adhesion within the epithelial layer.

These findings likely reflect context- and model-dependent roles of epithelial NLRP3. In inflamed, primed airways, Toll-like receptor (TLR) ligands, allergens, and cytokines have been shown to upregulate NLRP3 and pro-IL-1β, thereby facilitating inflammasome activation (81, 82). In such settings, the inhibition of NLRP3 has been observed to offer protective effects (79). Conversely, in minimally primed monolayers, basal NLRP3 may contribute to junctional homeostasis, suggesting that acute blockade can transiently reduce *Tjp-1* and *CDH1*. Varying results can also arise from differences between immortalized lines and well-differentiated primary human bronchial epithelial cells (HBECs), species and asthma endotypes, and from pharmacologic versus genetic disruptions (83). Furthermore, evidence suggests that epithelial-to-mesenchymal transition (EMT) and oxidative stress, driven by epidermal growth factor receptor (EGFR) and transforming growth factor beta (TGF−β), respectively, independently regulate tight junctions, providing an alternative explanation for the reduced expression of *Tjp-1* and *CDH1* following NLRP3 inhibition (84).

Combined with earlier studies that link NLRP3 expression to the integrity of the epithelial barrier, our findings indicate that NLRP3 is a crucial regulator of epithelial stability and barrier function. Importantly, we demonstrate here that naïve NLRP3^-/-^ mice exhibit intrinsic defects in tight junction expression and E-cadherin levels, leading to increased allergen uptake, a baseline phenotype not previously reported in asthma models. This role highlights NLRP3 as an essential factor in epithelial homeostasis and underscores its potential as a therapeutic target for interventions to improve barrier integrity in respiratory diseases, such as asthma. Therapeutic strategies targeting NLRP3 in asthma will need to consider its dual role in both inflammation and epithelial barrier homeostasis. Selective modulation of inflammasome-dependent versus homeostatic functions may allow preservation of epithelial integrity while limiting type 2 airway inflammation. Studies in human airway models will be crucial for translating these insights into effective clinical interventions. In comparison to previous studies that focused on the impact of NLRP3 on epithelial permeability and mucus production during inflammation, our results support the hypothesis that NLRP3 is integral to maintaining both the structural and functional properties of epithelial barriers across various tissues.

## Data Availability

The original contributions presented in the study are included in the article/**Supplementary Material**. Further inquiries can be directed to the corresponding author.

## Group member of CHAMP study group

Gesine Hansen, Stephanie DeStefano, Bianca Schaub, Erika von Mutius, Andreas Böck, Jana Kristin Eckert, Kathrin Urner, Kathrin Kalkbrenner, Franziska Sattler, Sabine Schnadt, Julia Kahle, Wolfgang Greiner, Simone Kreimeier, Ole Marten, Kirsten Beyer, Young-Ae Lee, Josefine Dobbertin-Welsch, Eckard Hamelmann, Christina Schorlemer, Michael Kabesch, Parastoo Kheiroddin, Martin Depner, Daniel Walter.
