# Supplementary material for: NLRP3 regulates epithelial barrier integrity and protects from airway hyperresponsiveness in experimental allergic asthma
Source: Front Immunol. 2025 Nov 27;16:1655205. doi: 10.3389/fimmu.2025.1655205 (PMC12695735; doi:10.3389/fimmu.2025.1655205)
Supplement: Supplementary file 1 [file DataSheet1.pdf]

Supplementary Material

1 Supplementary Figures and Tables

1.1 Supplementary Tables

Supplementary Table 1: Antibodies used for flow cytometric analysis

Antibody	Clone	Company
**Dendritic cells (DCs)**		
CD3e	145-2C11	BD
MHCII	M5/114.15.2	Invitrogen
SiglecF	REA798	MiltenyiBiotech
CD11b	M1/70	BioLegend
CD11c	N418	BioLegend
CD19	6D5	BioLegend
CD115	AFS98	BioLegend
**Epithelial cells**		
CD45	30-F11	BioLegend
CD31	MEC13.3	BioLegend
E-cadherin	DECMA1	BioLegend
CD326 (EpCAM)	G8.8	Invitrogen
Isotype (E-cadherin)	IgG1,k	BioLegend

Supplementary Table 2: Primer

**Gene**	**Gene ID**	**Assay ID**	**Assay type**	**Company**
**mouse**				
Muc5AC	Mm_Muc5ac_1_SG	QT01196006	QuantiTect	Qiagen
Tjp1	Mm_Tjp1_1_SG	QT00493899	QuantiTect	Qiagen
Cldn18	Mm_Cldn18_1_SG	QT00121142	QuantiTect	Qiagen
GAPDH	Mm_Gapdh_3_SG	QT01658692	QuantiTect	Qiagen
**human**				
Tjp1	Hs_TJP1_1_SG	QT00077308	QuantiTect	Qiagen
CDH1	Hs_CDH_1_SG	QT00080143	QuantiTect	Qiagen
GAPDH	Hs_GAPDH_1_SG	QT00079247	QuantiTect	Qiagen

1.2 Supplementary Figures

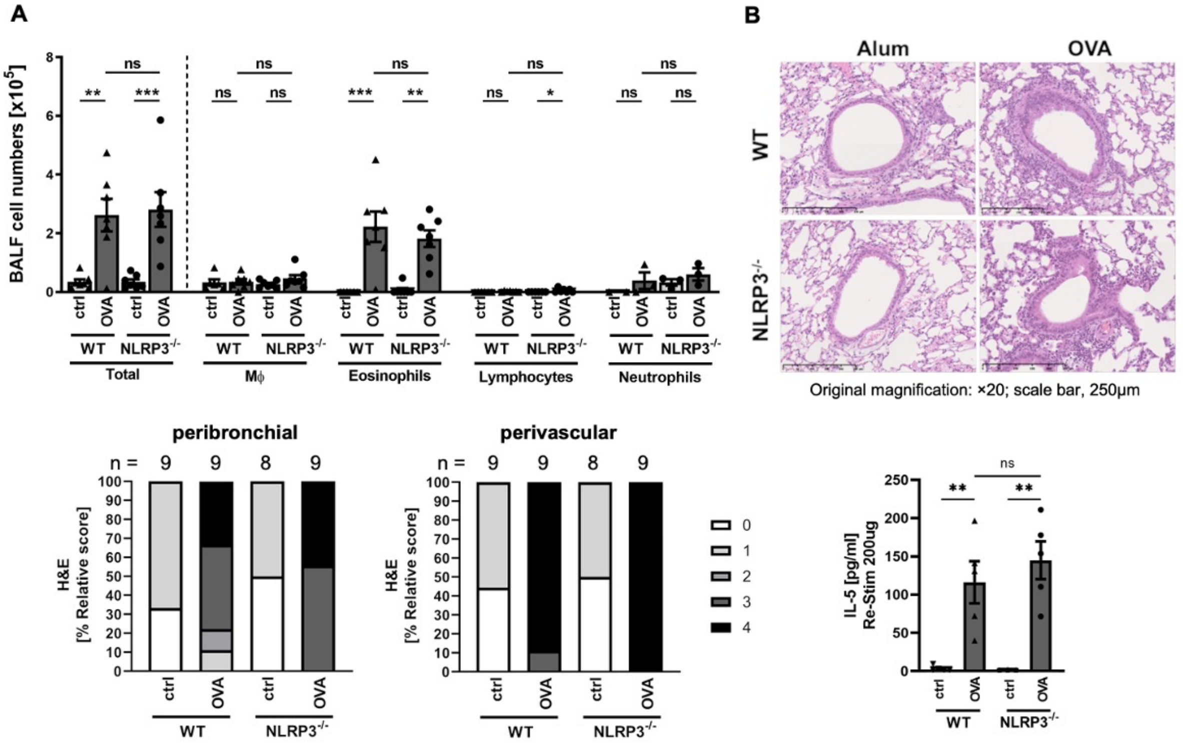

Supplementary Figure 1. Eosinophilic airway inflammation is comparable in OVA-immunized NLRP3$^{-/-}$ and WT mice. (A) Differential cell counts in the bronchoalveolar lavage fluid (BALF) from left to right: total BALF cell counts, macrophages, eosinophils, lymphocytes, and neutrophils; (each n≥3 mice/group) **(B)** Histological analysis of the inflammatory cell infiltration in H&E-stained lung sections from OVA-immunized WT and NLRP3$^{-/-}$ mice and controls. **(C)** Peribronchiolar and perivascular inflammation was quantified using a score from 0 to 4, where 0: no inflammation; 1: few cells; 2: a ring of inflammatory cells one-cell layer deep; 3: a ring of inflammatory cells two to four layers deep; and 4: a ring of inflammatory cells of more than four cell layers deep. Original magnification, ×20; scale bar, 200μm, representatives of n=8-9/group. White bars: alum, black bars: OVA. **(D)** Expression of IL-5 in supernatants of OVA re-stimulated lung cells determined by bead-based immunoassay (n≥5/group). One-way ANOVA; mean±SEM. ns not significant, * $P < 0.05$, ** $P < 0.01$, *** $P < 0.001$.

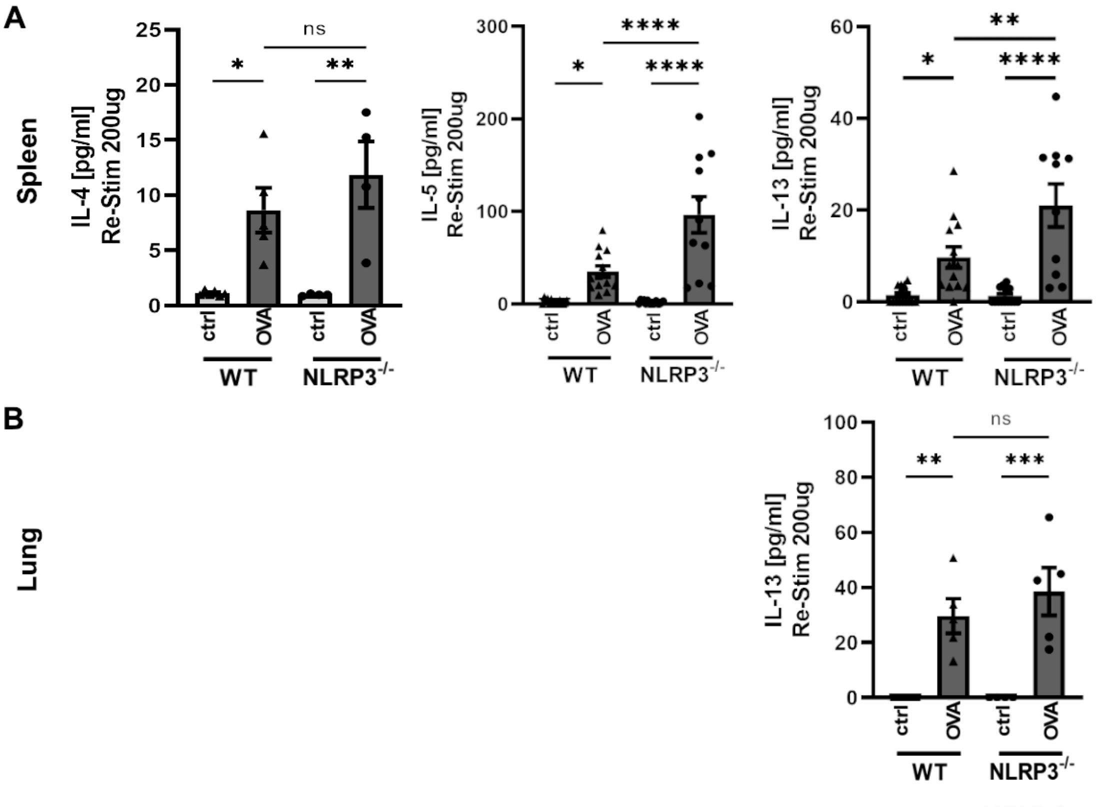

Supplementary Figure 2. Robust systemic cytokine expression in OVA-sensitized WT and NLRP3$^{-/-}$ mice. Expression of **(A)** IL-4, IL-5, and IL-13 in supernatants of OVA re-stimulated splenocytes, and **(B)** IL-13 in supernatants of OVA re-stimulated lung cells determined by bead-based immunoassay (n≥5/group). Add-on to **(A)**: IL-4 expression levels were below the detection limit; IL-5 expression is shown in Supplementary Figure 1. One-way ANOVA; mean±SEM. ns not significant, * $P < 0.05$, ** $P < 0.01$, *** $P < 0.001$ und **** $P < 0.0001$.
